# Medical Women

**Published:** 1898-07-09

**Authors:** 


### Modern Sociology.

### MEDICAL WOMEN.

The curious and interesting change which has come over the position of women during the latter half of the Queen's reign could hardly be more appropriately signalised than by the Royal sanction which will next week be given to the great experiment, as by many it has been considered, of granting medical diplomas to women, and giving them a legal status as qualified medical practitioners.

If we look back to the fierce conflict of opinion, which we may almost say raged a quarter of a century ago around the question, whether it was becoming or even decent for women, and especially for young women, to study medicine; and if we bear in mind that the point around which the opponents of the new departure always rallied when routed from every other position was the abomination of women and girls fresh from school opening their eyes to the "horrors" of the dissecting-room and opening their minds to the "disgusting details" of physiology, we can begin to appreciate the marvellous change in public opinion as to woman's proper share in the fruits of the tree of knowledge, which is indicated by the fact that the new science laboratories of the London School of Medicine for Women will next week be opened by the gracious Princess who stands in the eye of the British public as the representative of all that is pure and true in womanhood, Her Royal Highness the Princess of Wales.

Nor can it be said that the benefit of the altered view from which science is now regarded has fallen to woman alone. The root idea which made well-minded men shrink back at the thought of girls studying medicine, and led them to declare that they had rather see their daughters lying in their graves than studying anatomy in the dissecting-room, was no doubt the notion that there was something inherently demoralising both in the knowledge sought by such means and in the steps by which it was attained. It was held that, although it was necessary that doctors should know certain things, it was an evil; that such knowledge, even to man, implied something which detracted from his nobility; and that to impart such knowledge to woman was to take from her that tender bloom of virgin innocence by which she was imagined to be distinguished up to that mystic moment in her life when, with full canonical ceremonies, her finger was encircled by a hoop of gold, and the scales fell from her eyes.

We do not now believe less in the innocence of women, but we look upon virtue and continence as being alike quite independent of and separate from ignorance. Thus the recognition of medicine, and all that the word connotes, as an appropriate career for women, while to them it means the knocking down of a great barrier, and the removal of a great sign of separation and, as some would have it, of inferiority, at which a few of them have girded greatly and with much clamour, means something else to men, for if, without loss of delicacy and womanliness, all knowledge can be laid bare to the more tender sex, man must be acquitted of brutality in searching for knowledge how and where best it may be found.

Those who would appreciate the meaning of the change should read Dickens and Albert Smith as to the medical student of their days, and then look into some of the newer dissecting rooms at the best managed medical schools, or visit the school of human anatomy at the Oxford Museums, and then it will be seen what science does to render pure what is in itself perhaps repulsive, and to make it possible for girls to go without shrinking, always protected by the mantle of purity of intention, to their morning's work in what is understood to be "the prettiest" dissecting room in London. Surely, it is appropriate that these science laboratories should be opened by and should receive the sanction of one whose husband's motto is "Honi Soit Qui Mal y Pense." The story of the fight, the tale of the struggles, and the history of all that was done by the pioneers in the early days is not for us now to tell; but the fight was hard and the discouragement was great. Not only did the examining bodies mostly refuse to admit women to their examinations, but the schools were closed, so that those few ladies who first worked their way up to within reach of a diploma had to trust to a considerable extent to private teaching, not only in the more special scientific subjects, but in regard to all that is embraced under the name of hospital practice. This, of course, entailed great expense, and was practically prohibitive. Still, by degrees, the candidates grew in number, and so soon as qualifications were opened to them women were found ready enough to pass the examinations, and little by little one door after another has been opened until now we have not only a flourishing school of medicine for women in London, but two schools in Edinburgh (the Edinburgh School of Medicine for Women and the Medical College for Women), the Queen Margaret College in Glasgow, and the School of Surgery of the Royal College of Surgeons in Ireland, the first three named above being exclusively for women. At all of these a complete medical education can be obtained, but there are many other institutions where individual courses can be taken out.

As to qualifications, the University of London, the University of Durham, the University of Edinburgh, the University of Glasgow, the University of Aberdeen, and the Royal University of Ireland, the Society of Apothecaries, London, and the Conjoint Board of the Royal Colleges of Physicians and Surgeons in Ireland, all admit women as candidates for their degrees and diplomas, and women are subject to the regulations of the General Medical Council in precisely the manner as is the case in regard to men.

It is a great change, a great development, a great example of progress, on the attainment of which the pioneers may well feel gratification.

---

During the past month the National Society for the Prevention of Cruelty to Children investigated 2,510 complaints of neglect and ill-treatment, of which 2,191 were dealt with. The cases found true numbered 2,061, affecting the welfare of 5,686 children.

